# Supplementary material for: Physics-informed neural network for lithium-ion battery degradation stable modeling and prognosis
Source: Nat Commun. 2024 May 21;15:4332. doi: 10.1038/s41467-024-48779-z (PMC11109204; doi:10.1038/s41467-024-48779-z)
Supplement: Supplementary file 1 — Supplementary Information [file 41467_2024_48779_MOESM1_ESM.pdf]

Physics-informed neural network for lithium-ion battery degradation stable modeling and prognosis

Fujin Wang [a,b,*], Zhi Zhai[a,b,*], Zhibin Zhao[a,b,**], Yi Di[a,b], Xuefeng Chen[a,b,**]

[a] *National and Local Joint Engineering Research Center of Equipment Operation Safety and Intelligent Monitoring, Xi'an Jiaotong University, Xi'an, 710049, PR China*
[b] *School of Mechanical Engineering, Xi'an Jiaotong University, Xi'an, 710049, PR China*

*These authors contributed equally to this work.
**Corresponding authors, E-mail address: zhaozhibin@xjtu.edu.cn. chenxf@mail.xjtu.edu.cn. Telephone: +86 18717343671.

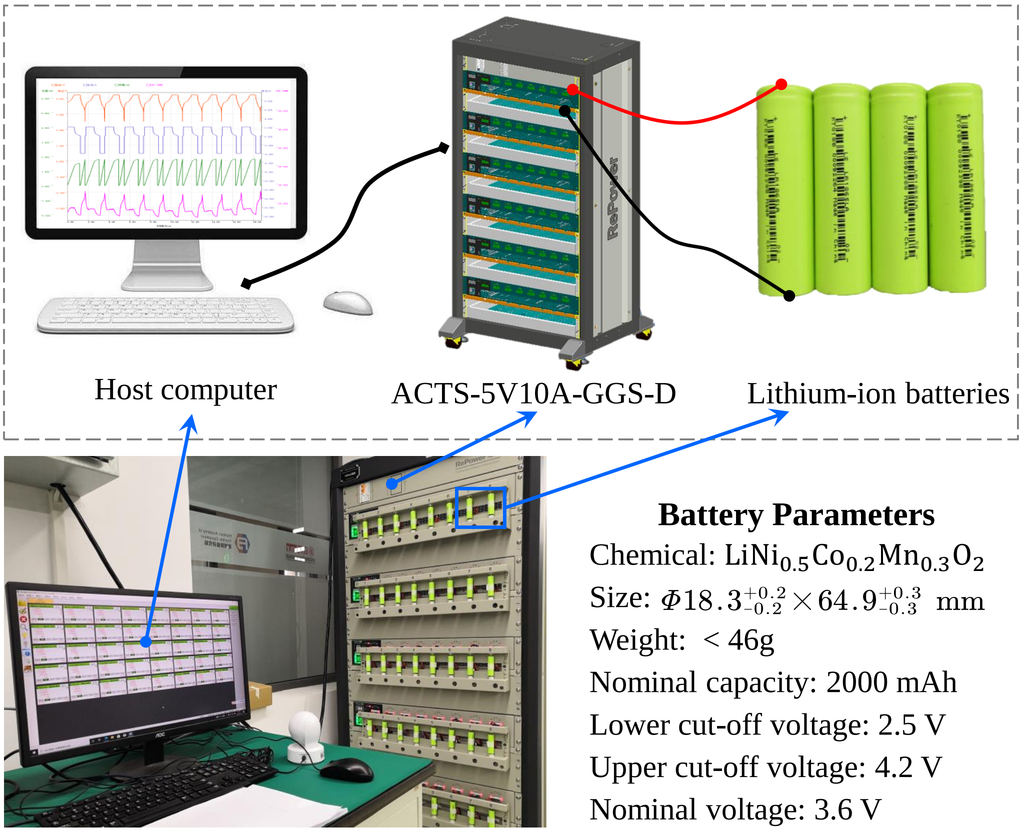

Figure S.1: The battery degradation platform in our laboratory. All batteries cycled to failure in 40-channel ACTS-5V10A-GGS-D at room temperature.

Supplementary Note 1. Dataset description

We use *batch 1* to *batch 6* to represent the 6 charging and discharging protocols, respectively. All batches except batch 2 consist of 8 batteries, while batch 2 contains 15 batteries. The voltage, current, temperature, and discharge capacity curves of the first battery in each batch are given in Figure S.4.

Supplementary Note 1.1. batch 1

The batteries in batch 1 were cycled under a fixed charging and discharging strategy. All batteries were charged to 4.2 V at 2 C with constant voltage and constant current (CC-CV) mode and then discharged to 2.5 V at 1 C.

Supplementary Note 1.2. batch 2

Batch 2 contains 15 batteries, and its charging and discharging strategy is similar to batch 1. All batteries were charged to 4.2 V at 3 C with CC-CV mode and then discharged to 2.5 V at 1 C.

Supplementary Note 1.3. batch 3

Batch 3 has a more complex protocol than that of the first two batches. All batteries were charged at 2 C with CC-CV mode. Then they were discharged to 2.5 V with a current value of x C, where $x \in \{0.5, 1, 2, 3, 5\}$.

Supplementary Note 1.4. batch 4

Batch 4 is similar to batch 3. The batteries were charged at 2 C with CC-CV mode and then discharged to 3.0 V with the same current as batch 3.

Supplementary Note 1.5. batch 5

Batch 5 follows the random walking strategy, thereby the entire process of charging and discharging are more closely with real-life usage. Specifically, all cells are charged to 4.2 V at 1 C with CC-CV mode and then discharge to 3.0 V. The discharge current is a random integer in the range of $[2, 8]$ ampere and the duration is in the range of $[2, 6]$ minutes.

Supplementary Note 1.6. batch 6

In batch 6, we simulated the charging and discharging strategy of a satellite in geosynchronous earth orbit (GEO). The batteries of GEO satellites only supply power during the shadow period of the earth, and the depth-of-discharge (DOD) is generally less than 80%. The

duration of each discharge is determined by the duration of the Earth's shadow. Therefore, the discharge duration of each cycle is different, and the DOD is also different.

Specifically, GEO satellites experience the Earth's shadow during the spring and autumn equinoxes each year, approximately 23 days before and after the equinoxes, resulting in a total duration of approximately 46 days for each occurrence. During the period of the Earth's shadow, the duration of the shadow varies in a regular pattern every day, as depicted in Table S.1 and Figure A.12. The duration of the shadow initially increases gradually before decreasing. An illustration of the discharge capacity curve of battery 1 in batch 6 is given in Figure S.3.

Table S.1: The discharge duration of each cycle of the GEO satellite in the Earth's shadow period [1].

Cycle number	1	2	3	4	5	6	7	8	9	10	11	12	13	14	15	16	17	18	19	20	21	22	23
	46	45	44	43	42	41	40	39	38	37	36	35	34	33	32	31	30	29	28	27	26	25	24
Discharge duration	5	20	34	41	46	50	54	56	58	60	62	64	68	69	70	71	72	72	72	72	72	72	72

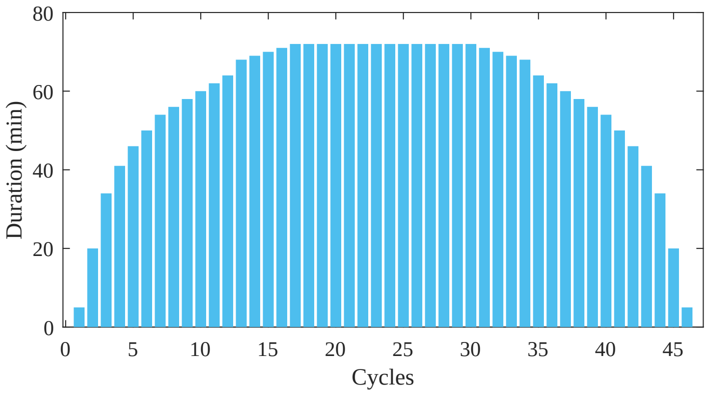

Figure S.2: The discharge duration of each cycle of the GEO satellite in the Earth's shadow period.

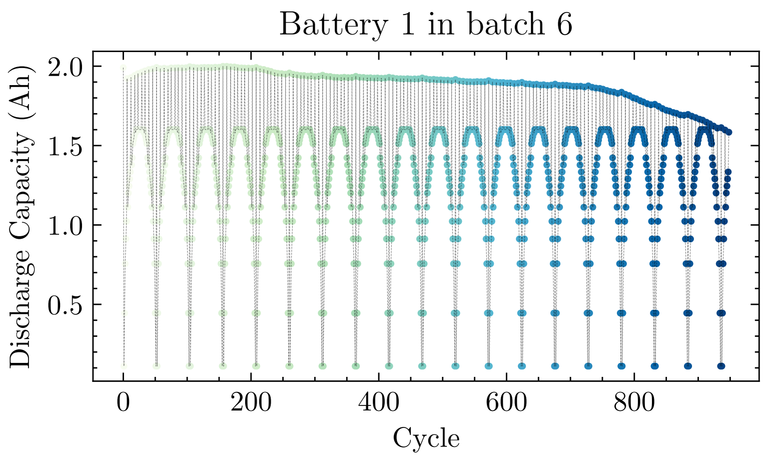

Figure S.3: An illustration of the discharge capacity curve of battery 1 in batch 6 during the whole life cycles.

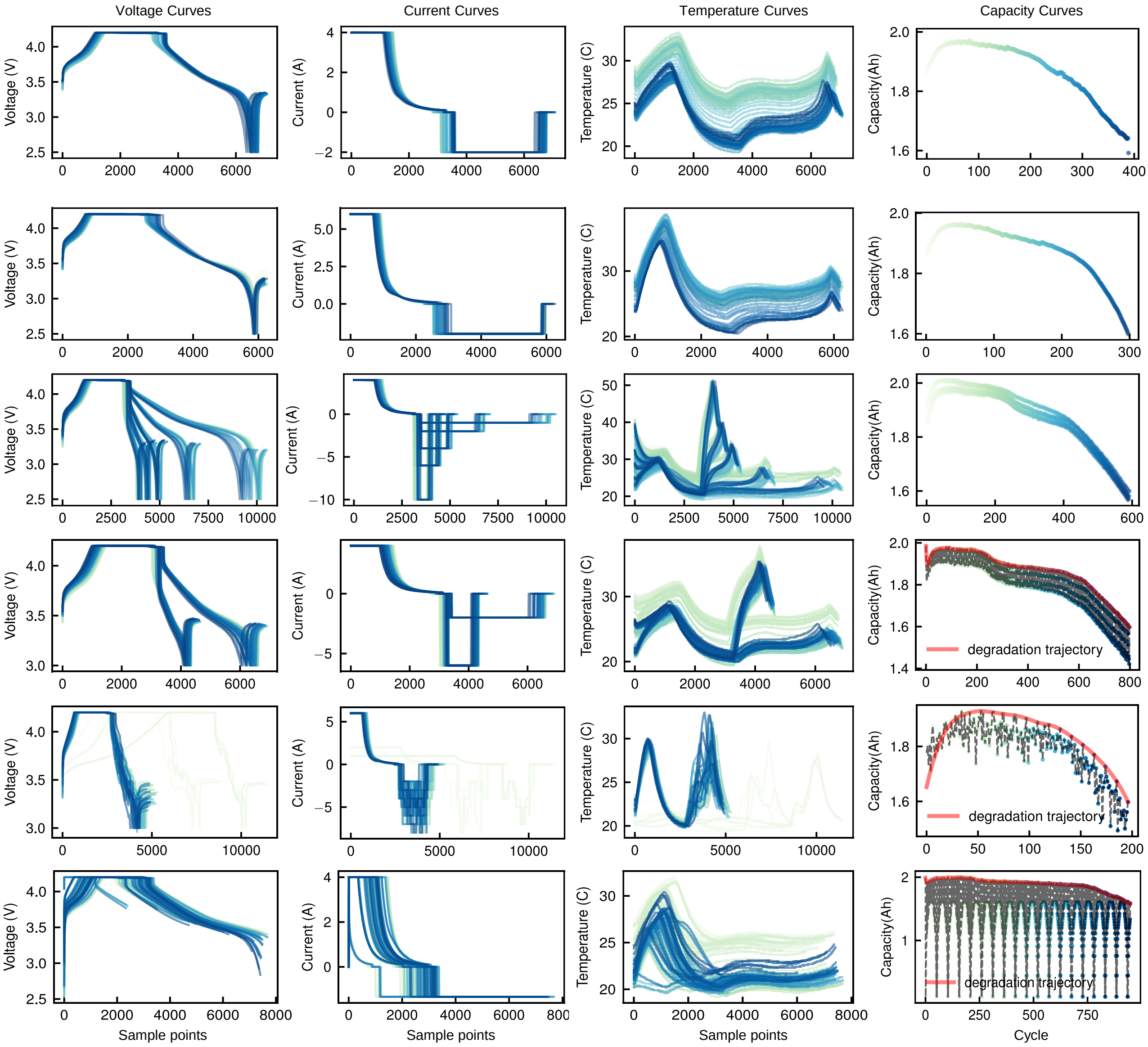

Figure S.4: The voltage, current, temperature, and discharge capacity curves during the whole life cycles of the XJTU battery dataset. Each row represents the curve of the first battery in the corresponding batch. From batch 3 to batch 6, the experimental settings are all incomplete discharge protocols, so we measure the discharge capacity after every few cycles, and finally interpolate the discharge capacity curve to obtain the red degradation trajectory curve in the figure.

Supplementary Note 2. Feature extraction

For the XJTU and TJU datasets, the voltage curve whose value is in the range [4.0, 4.2] V is selected, and for the MIT and HUST datasets, the voltage range is [3.4, 3.6] V. Suppose the time range corresponding to the selected data is $[t_{\text{start}}, t_{\text{end}}]$, the mean, standard deviation, kurtosis, skewness, charging time, accumulated charge, curve slope, and curve entropy are calculated as:

$$\bar{x} = \frac{1}{n}\sum_{i=1}^{n} \mathbf{x}(i), \tag{1}$$

$$\sigma = \sqrt{\frac{1}{n-1}\sum_{i=1}^{n} \left(\mathbf{x}(i) - \bar{x}\right)^2}, \tag{2}$$

$$\text{kurtosis} = \frac{\sum_{i=1}^{n} \left(\mathbf{x}(i) - \bar{x}\right)^4}{(n-1)\,\sigma^4}, \tag{3}$$

$$\text{skewness} = \frac{\sum_{i=1}^{n} \left(\mathbf{x}(i) - \bar{x}\right)^3}{(n-1)\,\sigma^3}, \tag{4}$$

$$\Delta t = t_{\text{end}} - t_{\text{start}}, \tag{5}$$

$$\Delta Q = \int_{t_{\text{start}}}^{t_{\text{end}}} I \cdot dt, \tag{6}$$

$$\text{slope} = \frac{V_{\text{end}} - V_{\text{start}}}{t_{\text{end}} - t_{\text{start}}}, \tag{7}$$

$$\text{entropy} = -\sum_{i=1}^{n} p_i \cdot \log\left(p_i\right), \tag{8}$$

where p_i is normalized value of a curve. For current curve with value between [0.5, 0.1] A, the features are calculated in the same way as above.

Table S.2: The number of test batteries in each dataset.

Dataset	Batch	Total number	Number of test set
XJTU	1	8	2
	2	15	3
	3	8	2
	4	8	2
	5	8	2
	6	8	2
TJU	1	66	13
	2	55	11
	3	9	2
HUST	-	77	20
MIT	-	125	23

Note: In the paper [2], the MIT dataset includes 124 batteries. In fact, we found that a total of 135 batteries were included in the 3 MATLAB files, because some of the data were not included in the paper [2] for some errors. In our study, we used a total of 125 batteries by removing some battery data that encountered errors during feature extraction. The battery ID is the number of the data in the MATLAB file. More details can be found in our code: code link.

Table S.3: The estimation errors of the proposed PINN (ours), multi-layer perceptron (MLP) and convolutional neural network (CNN) on the XJTU battery dataset. MAPE is the mean absolute percentage error and RMSE is the root mean square error. All values are averages from 10 experiments.

Batch	Battery	Ours		MLP		CNN	
		**MAPE**	**RMSE**	**MAPE**	**RMSE**	**MAPE**	**RMSE**
**1**	**battery-4**	0.0071	0.0105	0.0276	0.0295	0.0187	0.0240
	**battery-8**	0.0070	0.0084	0.0244	0.0259	0.0352	0.0419
**2**	**battery-4**	0.0145	0.0156	0.0310	0.0354	0.0359	0.0410
	**battery-8**	0.0106	0.0112	0.0331	0.0348	0.0243	0.0298
	**battery-14**	0.0087	0.0098	0.0185	0.0209	0.0290	0.0346
**3**	**battery-4**	0.0102	0.0120	0.0190	0.0222	0.0197	0.0232
	**battery-8**	0.0070	0.0080	0.0232	0.0251	0.0157	0.0192
**4**	**battery-4**	0.0063	0.0099	0.0185	0.0226	0.0140	0.0175
	**battery-8**	0.0080	0.0112	0.0215	0.0244	0.0160	0.0203
**5**	**battery-4**	0.0094	0.0113	0.0154	0.0174	0.0371	0.0447
	**battery-8**	0.0116	0.0157	0.0212	0.0260	0.0328	0.0460
**6**	**battery-4**	0.0081	0.0130	0.0225	0.0268	0.0150	0.0200
	**battery-8**	0.0046	0.0063	0.0184	0.0215	0.0148	0.0188

Table S.4: The estimation errors of the proposed PINN (ours), multi-layer perceptron (MLP) and convolutional neural network (CNN) on the TJU battery dataset. MAPE is the mean absolute percentage error and RMSE is the root mean square error. All values are averages from 10 experiments.

**Batch**	**Battery**	**Ours**		**MLP**		**CNN**	
		**MAPE**	**RMSE**	**MAPE**	**RMSE**	**MAPE**	**RMSE**
**1**	**CY25-025_1-#5**	0.0197	0.0180	0.0227	0.0211	0.0177	0.0178
	**CY25-05_1-#10**	0.0119	0.0124	0.0167	0.0165	0.0202	0.0207
	**CY25-05_1-#16**	0.0099	0.0101	0.0113	0.0116	0.0149	0.0159
	**CY25-05_1-#2**	0.0100	0.0098	0.0123	0.0122	0.0161	0.0172
	**CY25-05_1-#8**	0.0470	0.0436	0.0548	0.0489	0.0294	0.0325
	**CY25-1_1-#3**	0.0146	0.0154	0.0188	0.0202	0.0374	0.0395
	**CY25-1_1-#9**	0.0123	0.0139	0.0155	0.0174	0.0161	0.0183
	**CY45-05_1-#1**	0.0309	0.0264	0.0362	0.0310	0.0245	0.0234
	**CY45-05_1-#15**	0.0113	0.0111	0.0177	0.0170	0.0151	0.0158
	**CY45-05_1-#19**	0.0098	0.0093	0.0144	0.0139	0.0128	0.0125
	**CY45-05_1-#24**	0.0086	0.0086	0.0143	0.0139	0.0114	0.0118
	**CY45-05_1-#28**	0.0080	0.0080	0.0106	0.0109	0.0086	0.0089
	**CY45-05_1-#8**	0.0190	0.0185	0.0223	0.0213	0.0335	0.0358
**2**	**CY25-05_1-#12**	0.0234	0.0261	0.0236	0.0267	0.0179	0.0201
	**CY25-05_1-#16**	0.0133	0.0164	0.0144	0.0166	0.0143	0.0158
	**CY25-05_1-#21**	0.0198	0.0232	0.0271	0.0291	0.0281	0.0286
	**CY25-05_1-#4**	0.0190	0.0230	0.0231	0.0264	0.0232	0.0236
	**CY35-05_1-#1**	0.0119	0.0108	0.0135	0.0125	0.0105	0.0106
	**CY45-05_1-#1**	0.0089	0.0090	0.0125	0.0124	0.0132	0.0134
	**CY45-05_1-#15**	0.0099	0.0110	0.0114	0.0118	0.0160	0.0163
	**CY45-05_1-#19**	0.0043	0.0050	0.0065	0.0069	0.0085	0.0086
	**CY45-05_1-#24**	0.0047	0.0045	0.0077	0.0072	0.0075	0.0074
	**CY45-05_1-#28**	0.0042	0.0040	0.0094	0.0088	0.0066	0.0067
	**CY45-05_1-#8**	0.0111	0.0116	0.0151	0.0150	0.0121	0.0123
**3**	**CY25-05_2-#2**	0.0069	0.0070	0.0133	0.0133	0.0107	0.0110
	**CY25-05_4-#3**	0.0090	0.0088	0.0166	0.0156	0.0140	0.0141

Table S.5: The estimation errors of the proposed PINN (ours), multi-layer perceptron (MLP) and convolutional neural network (CNN) on the HUST battery dataset. MAPE is the mean absolute percentage error and RMSE is the root mean square error. All values are averages from 10 experiments.

Battery	Ours		MLP		CNN	
	MAPE	RMSE	MAPE	RMSE	MAPE	RMSE
**1–4**	0.0070	0.0082	0.0071	0.0088	0.0070	0.0086
**1–8**	0.0061	0.0065	0.0070	0.0076	0.0061	0.0067
**2–4**	0.0078	0.0083	0.0073	0.0080	0.0073	0.0082
**2–8**	0.0053	0.0062	0.0049	0.0059	0.0073	0.0088
**3–4**	0.0080	0.0096	0.0090	0.0104	0.0104	0.0121
**3–8**	0.0103	0.0110	0.0112	0.0124	0.0098	0.0107
**4–4**	0.0084	0.0091	0.0079	0.0088	0.0073	0.0083
**4–8**	0.0090	0.0103	0.0090	0.0103	0.0077	0.0090
**5–4**	0.0056	0.0068	0.0063	0.0075	0.0050	0.0061
**5–7**	0.0053	0.0064	0.0051	0.0063	0.0058	0.0068
**6–4**	0.0119	0.0124	0.0115	0.0121	0.0098	0.0116
**6–8**	0.0123	0.0132	0.0125	0.0137	0.0112	0.0145
**7–4**	0.0058	0.0068	0.0067	0.0079	0.0063	0.0072
**7–8**	0.0069	0.0075	0.0077	0.0082	0.0063	0.0074
**8–4**	0.0056	0.0068	0.0053	0.0064	0.0069	0.0083
**8–8**	0.0099	0.0106	0.0110	0.0117	0.0074	0.0082
**9–4**	0.0145	0.0149	0.0145	0.0150	0.0102	0.0114
**9–8**	0.0036	0.0042	0.0033	0.0040	0.0046	0.0055
**10–4**	0.0090	0.0105	0.0084	0.0097	0.0068	0.0085
**10–8**	0.0035	0.0042	0.0042	0.0051	0.0047	0.0056

Table S.6: The estimation errors of the proposed PINN (ours), multi-layer perceptron (MLP) and convolutional neural network (CNN) on the MIT battery dataset. MAPE is the mean absolute percentage error and RMSE is the root mean square error. All values are averages from 10 experiments.

Date	Battery	Ours		MLP		CNN	
		MAPE	RMSE	MAPE	RMSE	MAPE	RMSE
2017/5/12	battery-5	0.0091	0.0109	0.0044	0.0050	0.0069	0.0077
	battery-10	0.0032	0.0037	0.0059	0.0070	0.0057	0.0062
	battery-15	0.0069	0.0073	0.0053	0.0057	0.0070	0.0077
	battery-20	0.0036	0.0040	0.0068	0.0075	0.0065	0.0070
	battery-25	0.0073	0.0076	0.0038	0.0047	0.0044	0.0053
	battery-30	0.0038	0.0046	0.0028	0.0033	0.0036	0.0042
	battery-35	0.0034	0.0037	0.0062	0.0065	0.0057	0.0062
	battery-40	0.0043	0.0046	0.0090	0.0098	0.0059	0.0068
	battery-45	0.0062	0.0073	0.0099	0.0124	0.0096	0.0129
2017/6/30	battery-5	0.0104	0.0103	0.0023	0.0027	0.0039	0.0045
	battery-15	0.0022	0.0026	0.0243	0.0240	0.0163	0.0173
	battery-20	0.0183	0.0187	0.0126	0.0194	0.0086	0.0132
	battery-25	0.0093	0.0155	0.0055	0.0059	0.0036	0.0042
	battery-30	0.0056	0.0060	0.0080	0.0096	0.0078	0.0098
	battery-35	0.0072	0.0093	0.0044	0.0051	0.0040	0.0048
	battery-40	0.0041	0.0049	0.0073	0.0080	0.0041	0.0048
	battery-45	0.0059	0.0067	0.0139	0.0133	0.0055	0.0060
2018/4/12	battery-10	0.0040	0.0053	0.0055	0.0061	0.0049	0.0059
	battery-15	0.0069	0.0070	0.0084	0.0084	0.0057	0.0062
	battery-25	0.0106	0.0106	0.0099	0.0100	0.0121	0.0123
	battery-30	0.0021	0.0024	0.0033	0.0037	0.0026	0.0031
	battery-40	0.0124	0.0122	0.0133	0.0130	0.0109	0.0109
	battery-45	0.0067	0.0075	0.0083	0.0086	0.0053	0.0064

Table S.7: The results of 3 models (the proposed PINN (Ours), multilayer perceptron (MLP), and convolutional neural network (CNN)) in small sample experiments on XJTU battery dataset. MAPE is mean absolute percentage error and RMSE is root mean square error. All values are averaged from 10 experiments.

**Batch**	**Train Batteries**	**Ours**		**MLP**		**CNN**	
		**MAPE**	**RMSE**	**MAPE**	**RMSE**	**MAPE**	**RMSE**
**1**	**1**	0.0141	0.0184	0.0343	0.0390	0.0929	0.0949
	**2**	0.0105	0.0134	0.0267	0.0304	0.0728	0.0826
	**3**	0.0069	0.0096	0.0347	0.0383	0.0548	0.0666
	**4**	0.0056	0.0076	0.0292	0.0327	0.0560	0.0647
**2**	**1**	0.0954	0.0516	0.5480	0.1204	3.3605	0.2915
	**2**	0.0197	0.0262	0.0648	0.0629	0.2045	0.1469
	**3**	0.0106	0.0119	0.0264	0.0308	0.1066	0.1165
	**4**	0.0115	0.0130	0.0284	0.0329	0.0744	0.0862
**3**	**1**	0.0240	0.0261	0.0336	0.0379	0.1579	0.1520
	**2**	0.0096	0.0112	0.0270	0.0312	0.0551	0.0659
	**3**	0.0090	0.0105	0.0273	0.0304	0.0374	0.0450
	**4**	0.0088	0.0102	0.0291	0.0324	0.0277	0.0340
**4**	**1**	0.0168	0.0199	0.0261	0.0301	0.1075	0.1130
	**2**	0.0075	0.0103	0.0218	0.0251	0.0623	0.0726
	**3**	0.0076	0.0106	0.0222	0.0251	0.0344	0.0427
	**4**	0.0070	0.0097	0.0205	0.0230	0.0238	0.0299
**5**	**1**	0.2519	0.1479	0.6330	0.1553	0.4337	0.2315
	**2**	0.0141	0.0184	0.0306	0.0392	0.1193	0.1210
	**3**	0.0157	0.0189	0.0303	0.0390	0.1084	0.1136
	**4**	0.0132	0.0168	0.0264	0.0349	0.1133	0.0971
**6**	**1**	0.0103	0.0135	0.0282	0.0351	0.2007	0.1577
	**2**	0.0096	0.0120	0.0196	0.0231	0.0499	0.0626
	**3**	0.0061	0.0088	0.0218	0.0264	0.0341	0.0437
	**4**	0.0072	0.0107	0.0205	0.0241	0.0247	0.0319

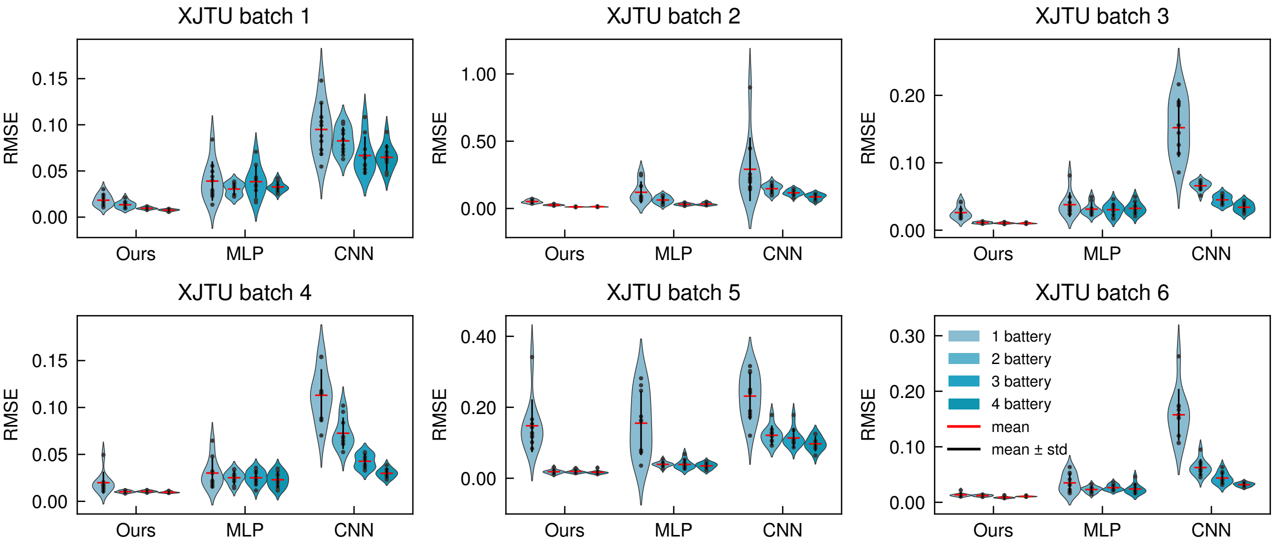

Figure S.5: An illustration of test root mean square error (RMSE) distributions for 3 models (the proposed PINN (Ours), multi-layer perceptron (MLP), and convolutional neural network (CNN)) on XJTU battery dataset. Each error bar contains 10 points (10 experiment) and is marked with mean and standard deviation lines. The legends have been added only on the last subplot. The "1 battery" in the legend means that we only use the data of 1 battery to train the model. Others are similar. As the number of batteries increases, the performance of the 3 models is getting better. However, our method still performs best among them.

Supplementary Note 3. Physics-informed neural network

Table S.8: The details of proposed PINN, multi-layer perceptron (MLP), and multi-layer perceptron (CNN). Sin refers to the sine function. BasicBlack is similar to that in ResNet [3], which consists of Conv1d, BatchNorm1d, ReLU, Conv1d, and BatchNorm1d.

Model	Module	Layer	Input size	Output size	Inference Param. Num.	Inference time/1000 sample
PINN	$\mathcal{F}(\cdot)$	Linear+Sin	17	60	7781	5.81e-04
		Linear+Sin	60	60		
		Linear	60	32		
		Linear+Sin	32	32		
		Linear	32	1		
	$\mathcal{G}(\cdot)$	Linear+Sin	35	60		
		Linear+Sin	60	60		
		Linear	60	1		
MLP		Linear+Sin	17	60	7781	5.64e-04
		Linear+Sin	60	60		
		Linear	60	32		
		Linear+Sin	32	32		
		Linear	32	1		
CNN		BasicBlock	(1,17)	(8,17)	8465	1.29e-02
		BasicBlock	(8,17)	(16,9)		
		BasicBlock	(16,9)	(24,5)		
		BasicBlock	(24,5)	(16,5)		
		BasicBlock	(16,5)	(8,5)		
		Linear	8*5	1		

Note: The values in the "Inference time/1000 sample" column represents the the time, in seconds, spent in inference per 1000 samples. Specifically, we set the batch size to 1000, count the time spent on 1000 forward inferences, and then take the average. Since the number of parameters of all three model is small, we do not use GPU for acceleration. Three models were implemented in Pytorch 1.7.1 on Intel Core i5-10400F CPU @ 2.90 GHz.

The parameters and structure of the proposed PINN, MLP, and CNN are given in Table S.8. The proposed PINN was learned by minimizing the loss defined in Equation (9) of the Manuscript File, and the Adam optimizer was used in the training phase. In the process of hyperparameter tuning, the Grid Search strategy was adopted to optimize the hypterparameter, including the number of PINN layers, the number of neurons in each layer, and α and β. The trade-off parameters α and β are set to 0.7 and 20 for XJTU battery dataset, 1 and 50 for TJU and MIT datasets, and 0.5 and 80 for HUST dataset. We set batch size to 256 for XJTU battery dataset and 512 for other 3 datasets. More details can be found in our codes.

References

[1] D. Zhao, Z. Zhou, S. Tang, Y. Cao, J. Wang, P. Zhang, Y. Zhang, Online estimation of satellite lithium-ion battery capacity based on approximate belief rule base and hidden markov model, Energy 256 (2022) 124632.

[2] K. A. Severson, P. M. Attia, N. Jin, N. Perkins, B. Jiang, Z. Yang, M. H. Chen, M. Aykol, P. K. Herring, D. Fraggedakis, et al., Data-driven prediction of battery cycle life before capacity degradation, Nature Energy 4 (5) (2019) 383–391.

[3] K. He, X. Zhang, S. Ren, J. Sun, Deep residual learning for image recognition, in: Proceedings of the IEEE conference on computer vision and pattern recognition, 2016, pp. 770–778.
